# Responsible robotics design–A systems approach to developing design guides for robotics in pasture-grazed dairy farming

**DOI:** 10.3389/frobt.2022.914850

**Published:** 2022-07-15

**Authors:** C. R. Eastwood, B. Dela Rue, J. P. Edwards, J. Jago

**Affiliations:** DairyNZ Ltd., Lincoln, New Zealand

**Keywords:** dairy, responsible innovation, co-design, transitions, socio-cyber-physical system

## Abstract

Application of robotics and automation in pasture-grazed agriculture is in an emergent phase. Technology developers face significant challenges due to aspects such as the complex and dynamic nature of biological systems, relative cost of technology versus farm labor costs, and specific market characteristics in agriculture. Overlaying this are socio-ethical issues around technology development, and aspects of responsible research and innovation. There are numerous examples of technology being developed but not adopted in pasture-grazed farming, despite the potential benefits to farmers and/or society, highlighting a disconnect in the innovation system. In this perspective paper, we propose a “responsibility by design” approach to robotics and automation innovation, using development of batch robotic milking in pasture-grazed dairy farming as a case study. The framework we develop is used to highlight the wider considerations that technology developers and policy makers need to consider when envisaging future innovation trajectories for robotics in smart farming. These considerations include the impact on work design, worker well-being and safety, changes to farming systems, and the influences of market and regulatory constraints.

## 1 Introduction

Robotics and automation technologies are having sector-wide impacts, such as in manufacturing, warehousing, and logistics. The application of such technologies in agriculture is still emerging, nevertheless there have been expectations that robotics could help farmers address staff shortage issues (Ryan et al., 2021), create more sustainable systems (Rose et al., 2021a), and improve productivity (Ingram et al., 2022). However, there are major barriers facing the use of robotics in agricultural systems, with limited uptake across agriculture globally (Lowenberg-DeBoer and Erickson, 2019; Eastwood et al., 2021). One issue, particularly in respect to Responsible Research and Innovation (RRI), is that smart farming technology development and promotion is often driven by technology developers, marketing, policy signals, and techno-centric portrayal of future agriculture in the media, rather than in direct response to defined farmer/producer requirements (Barrett and Rose, 2020; Lajoie-O'Malley et al., 2020). Additionally, aside from the core technical challenges associated with robotics and automation, agricultural systems present a challenging working environment for such technologies due to the dynamic nature of biological and climatic systems. The wide range of farm system contexts globally, for example different feeding systems for dairy cattle, can also impact market size for new technologies thereby limiting R&D investment (Eastwood et al., 2021).

Recent research into the topic of robotics, automation, and smart farming have identified some research and development challenges. A review by Ingram et al. (2022) identified priority research questions in agriculture, including the need to understand factors influencing the uptake of digital technology, how to include farmer perspectives in design of digital tools, and anticipating potential effects of such technologies on agriculture and agricultural work. Ryan et al. (2021) note research themes around benefit distribution of robots in agriculture, potential impacts on the idea of the “good farmer”, how robots can contribute to positive animal welfare, and the duality of enacting sustainable farming with robots. The interaction between robots and work have been explored in several studies (Legun and Burch, 2021; Nazareno and Schiff, 2021; Rose et al., 2021a). Additionally, Ditzler and Driessen (2022) state that while robotics is envisaged to “transform open-field agriculture”, a rethinking is required to broaden the robotic future imaginations to include more diverse farm systems and to incorporate aspects of sustainability. Finally, studies by Legun and Burch (2021) and Klerkx et al. (2019) identify the need to carefully examine the transitions to a robotic future, to be sensitive to the wider context (socio-economic, cultural and political), and to acknowledge that robotics represents just one possible transition pathway for future agricultural systems.

In this perspective paper, we consider the concepts of responsible innovation, systems thinking, and co-design to propose a design guide for development of robotics in pasture-grazed dairy farming. The paper builds on recent work interconnecting these domains by McCampbell et al. (2021), Eastwood et al. (2021), Rose et al. (2021a), Ryan et al. (2021), and Ditzler and Driessen (2022). Here, we address two questions related to the future use of robotics and automation systems in pasture-grazed dairy systems: 1) What are the key design criteria for development of robotics on pasture-grazed dairy systems to enable successful and responsible innovation? 2) What are potential implications of robotics for farm workplaces? We first develop a novel conceptual framework for design criteria for smart farming robotics, and then apply this framework to a robotic milking case study. The insights from this paper contribute to improved agricultural technology design and innovation practices.

## 2 Conceptual framework

### 2.1 Responsible innovation

The concept of RRI (Stilgoe et al., 2013) has been discussed in relation to various agricultural innovation contexts such as Agriculture 4.0 and smart farming (Rose and Chilvers, 2018; van der Burg et al., 2019; Eastwood et al., 2021), autonomous robotics (Rose et al., 2021a), the use of artificial intelligence (Smith, 2020), and cyber-physical systems (Rijswijk et al., 2021). RRI aims to include consideration of social values in development of technology and innovation through the attributes of anticipation, inclusion, reflexivity, and responsiveness (Eastwood et al., 2019b).

There has been limited work focused on the *anticipation* of farm system-level implications of robotics and automation in agriculture (Legun and Burch, 2021; Rose et al., 2021b; Ryan et al., 2021). We propose that part of the anticipatory process is to lead innovation through design with a systems-thinking lens (Romera et al., 2020; Eastwood et al., 2022). Identifying “design guides”, the priority features for a successful robot/agricultural system interface, is a potential approach to responsible design (Hess et al., 2021). Design guides could be aimed at broadening the innovation context to beyond just a techno-centric viewpoint, to acknowledge the wider context involving socio-ethical, farm systems, and innovation systems factors. The guides therefore provide an artefact around which RRI functions such as anticipation and reflexivity can be addressed (Eastwood et al., 2019b; Legun and Burch, 2021).

### 2.2 A framework for assessing responsible design of robotics in smart farming

Undertaking agricultural research and innovation based on a “ground-up” understanding of on-farm needs and challenges underpins many participatory approaches. The concept of design thinking is increasingly popular as a participatory approach in agriculture, to include end-users (farmers and stakeholders) by first understanding the “problem” and to understand the underlying context for successful innovation (Prost, 2021; Ditzler and Driessen, 2022; Eastwood et al., 2022). We propose a design guide, built on recent literature on robotics and automation in agriculture (see below), to facilitate this contextualization phase. The resulting 10 design considerations for robotics and automation in agriculture are: Farm operating systems, Workplace design and people, Farm business structure, Financial, Sustainability, Market factors, Social well-being, Regulation and policy, Knowledge base and networks, Technology and engineering (Table 1).

**TABLE 1:** Proposed design guide and considerations for responsible research and innovation into robotics and automation in smart farming.

Factor	Description of design considerations (with relevant references)
1 Farm operating systems	Key farm system features influencing technology use—e.g. *cows predominantly grazed on pasture, seasonal calving, feed sources used, mating or breeding processes used -* Lunner-Kolstrup et al. (2018)
	Alternative farm system configurations—such as solving labor shortages by combining work design changes and new technology
2 Workplace design and people	Impact (positive and negative) of technology on people (farmers and employees): Job context—*Workplace safety and conditions, Well-being and job satisfaction.* Job content—*Manual/cognitive tasks, Autonomy, Workload intensity* Lunner-Kolstrup et al. (2018), Nazareno and Schiff (2021), Rose et al. (2021a), Hansen et al. (2020), Klerkx et al. (2019)
3 Farm business structure	Influence of farm demographics—e.g., Farm size, life stage of existing infrastructure, current debt constraints on large capital investment, farmer career start (growing, stable, succession/exit)
	Farm business structure - influencing who makes the capital investment and who obtains value from technology—e.g., ownership structure (owner, lessee, sharefarmer), differences under capital purchase v subscription models
	Shifts in power balance between farmer and technology company, trust, human-robot interactions—Pigford et al. (2018), Ingram et al. (2022), Rose et al. (2021a), Basu et al. (2020)
4 Financial	Factors include the required capital investment (purchase v lease/subscription agreements), return on investment, and cost of alternative approaches, resale or depreciation rates—Renwick et al. (2017)
5 Sustainability	Innovations need to have a net positive impact on sustainability outcomes, including: Animal—*animal care, disease management*; Environment - *Nutrient and soil management, greenhouse gas emissions*—Renwick et al. (2017), Rose et al. (2021a)
6 Market factors	Influencing factors for technology design include: Market scale, supply chain factors, capturing value, end-user uncertainty, attitudes of farmers toward service costs for high tech equipment, impact of land prices, social and consumer perceptions—Renwick et al. (2017), Brier et al. (2020)
7 Social well-being	The wider impact on communities from changes in rural employment structures, community/consumer acceptance, equality, impact on farmer/farming identity—Renwick et al. (2017), Legun and Burch (2021), Vik et al. (2019)
8 Regulation and policy	Impacts of regulation related to water and environment, animals, health and safety, infrastructure, patent restrictions, food quality regulations—Renwick et al. (2017), Rose et al. (2021a)
9 Knowledge base and networks	The required changes in advisory knowledge, peer-to-peer support amongst farmers, confidence in technology across actors in the sector, technological integration or lock-in issues at sector level. Development of service and support networks.—Renwick et al. (2017), Brier et al. (2020), Eastwood et al. (2019a)
10 Technology and engineering	Technological performance, integration with other technologies, market uncertainty, data management and standards—Lunner-Kolstrup et al. (2018), Eastwood and Renwick (2020), Brier et al. (2020), Basu et al. (2020)

Insights from a range of literature were used to identify these 10 design guide factors. For example, the smart farming framework outlined in Eastwood et al. (2019a) and Brier et al. (2020) was initially developed as a technology assessment tool specifically for smart farming innovation. That framework incorporates aspects of target market characteristics, technology design and innovation, and capability requirements that are also relevant to robotics and automation. The need to consider a broad context when assessing potential agricultural innovation was identified by Renwick et al. (2017), including: aspects of financial performance, market factors, social well-being, environment, required knowledge base, and regulation. These insights helped to form the design guide factors of *Farm operating systems, Farm business structure, Financial, Sustainability, Market factors,* and *Regulation and policy*.

Specific challenges and opportunities associated with autonomous systems in agriculture were outlined by Rose et al. (2021a). Their analysis included themes of Renwick et al. (2017) while also including aspects of data use and management, trust in technology, and structural changes to agricultural communities through greater use of robotics. Research by Lunner-Kolstrup et al. (2018) also examined the technological functions of on-farm automation, data utilization by farmers, and the fit of automation with farming systems. Increased use of robotics has potential implications for power dynamics between farmers and technology providers, and these impacts need to be better anticipated in the design process (Pigford et al., 2018; Ingram et al., 2022). Many of these factors increase the innovation uncertainty related to a new technology, including uncertainty about technology performance, support networks, regulation and policy settings (Eastwood and Renwick, 2020). The implications of issues around data standards, data sharing, enabling infrastructure such as high-speed Internet, and even liability related to use of robotics are still uncertain in the agricultural context (Basu et al., 2020). These insights helped to form the design guide factors of *Knowledge base and networks* and *Technology and engineering*.

Robotics and automation have potentially significant positive and negative impacts on the agricultural work environment (Nazareno and Schiff, 2021; Rose et al., 2021a). Automating more manual, repetitive, and mundane tasks could help to retain people on farms (e.g., older farmers) while also making farms more attractive to new generations. Some argue that human well-being (physical, psychological, and social) should be the core component of the design process for technology development (Kafaee et al., 2021). The value of this lens was highlighted by Prause (2021) who found that some technologies can monitor employee performance and efficiency, potentially leading to efficiency gains but increased workplace stress and fatigue. We drew on these insights for the design guide factors of *Workplace design and people* and *Social well-being*.

## 3 Case study: High throughput robotic milking systems

### 3.1 Description of a high throughput robotic milking system concept

To illustrate application of the design guide process, we use a case study relevant to robotics and automation in pasture-based dairy farming, namely high throughput robotic milking systems. We define high throughput as milking systems that facilitate milking at a cows/hour throughput similar to existing batch milking systems, herringbone and rotary (e.g., 300 cows/hour). In the sections below we discuss potentially relevant opportunities and challenges associated with the factors identified in Table 1.

Robotic, or Automated Milking Systems (AMS), have been used on commercial dairy farms for almost 30 years (Rodenburg, 2017). The most common configuration is single “box” units, milking one cow at a time, and these systems have been widely adopted in European dairying countries and installations are increasing in North America (Vik et al., 2019; Eastwood and Renwick, 2020; Hansen et al., 2020). However, uptake of this style of AMS has been limited in grazed dairy systems such as in New Zealand and Australia (Eastwood and Renwick, 2020). There is still an appetite for novel milking robotics that fit with pasture-grazed systems, driven by difficulties attracting and retaining staff, a push for greater workforce productivity, to reduce wear and tear on workers and the undesirable nature of long days and “unsociable” hours on farm.

Major factors have hampered the success of single stall “box-style” robotic milking in large pasture-grazed dairy systems. These include: the large herd sizes relative to housed systems where robotic milking is most commonly used, batch milking systems based on twice a day milking, high throughput milking (e.g., 300 cows/hour) currently achieved in these systems, long distances and variable farm topography for cows to walk to milking facilities, business models built around low farm working expenses (due to variable pasture supply), and low labor input on a per cow basis. These factors combine to limit the financial viability of box-style robotic milking when compared to conventional milking systems in such large pasture systems (Eastwood and Renwick, 2020). Additionally, current commercially available “robotic rotary parlor” milking systems (from DeLaval and GEA) have seen limited adoption in Australia, and no installations to date in NZ. To fit this context, a robotic technology suited to high throughput batch milking is required.

We propose that a novel approach for high throughput robotic milking in this context would need to fit within general system constraints such as enabling batch milking, fit with larger herds (e.g., 400 + cows) using a block calving system, maximum 24 h between each milking, one person at the parlor in primarily a supervisory role, limited time where the herd is out of paddock, and maximum hours of work limited to a 12 h work day (e.g., 5 a.m.-5 p.m.). Overlaying these factors is the need for any new technology to have a positive value proposition for farmers.

### 3.2 Design considerations for high throughput robotic milking in pasture grazed systems

**FIGURE 1:**
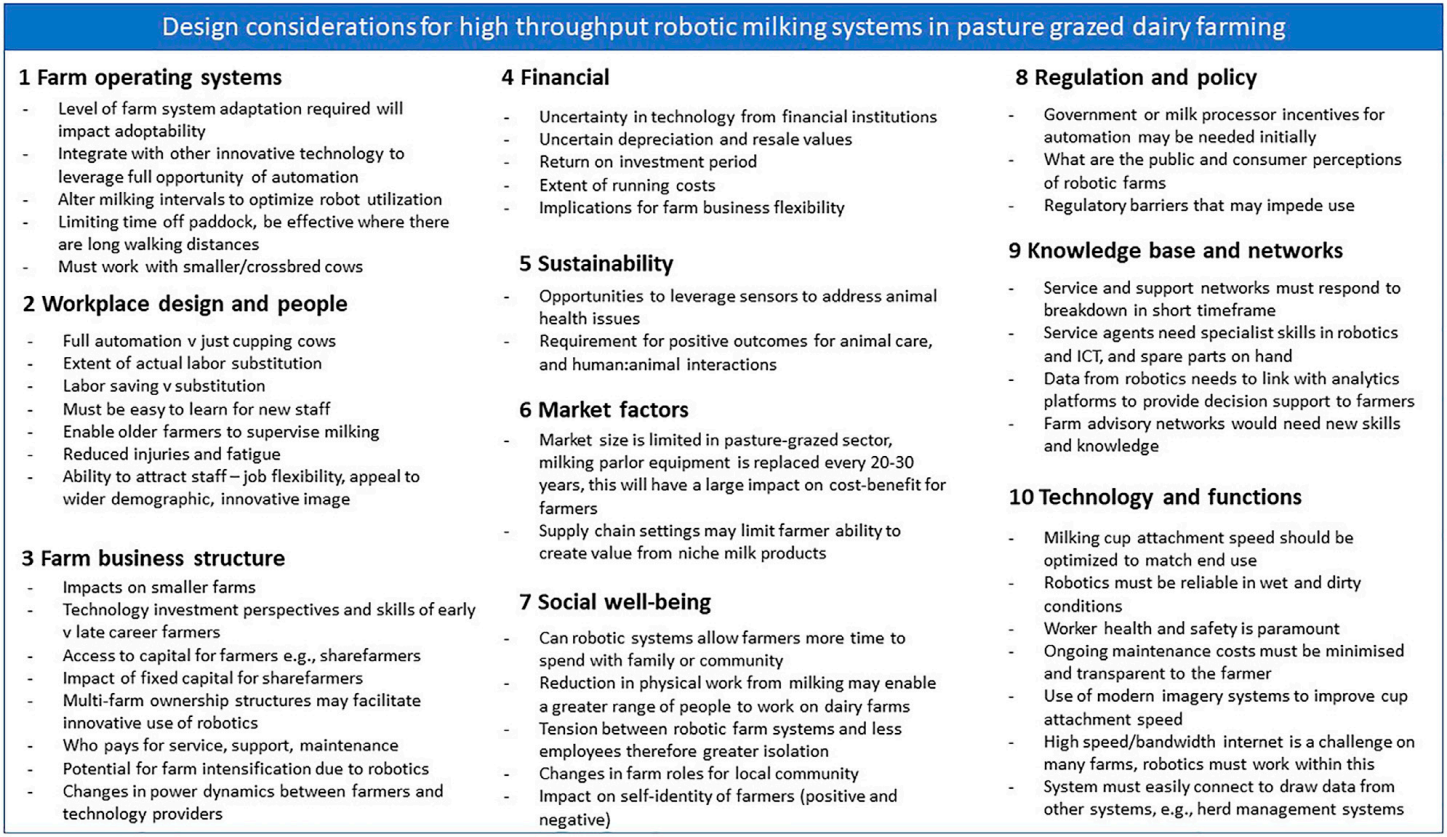
Design canvas highlighting design considerations related to developing high throughput robotic milking systems for NZ pasture grazed dairy farming.

In this section, we outline system design considerations for high throughput robotic milking systems in pasture grazed dairy farming, based on the framework outlined in Table 1. The potential design considerations are depicted in Figure 1 and explained further below.

#### 3.2.1 Farm operating systems

The extent of farm system adaptation required to incorporate milking robotics is highly influential, for example a retrofitted robot to cup cows via batch milking in existing rotary parlors would be much more palatable to farmers, compared with a totally new parlor requiring changes to the wider farm system (e.g., voluntary milking). The use of mobile robotic milking systems have also been trialed in grazed systems (Oudshoorn, 2008). Linking with other technology on farm, such as virtual fencing, could also leverage new opportunities for productivity gains–such as autonomously running multiple herds within a farm to utilize robotic milking throughout the day. Adapting milking intervals (e.g., once-a-day milking) could also be used to increase cow/robot capacity. A key component in pasture-grazed systems is also the need to limit the time the herd off paddock for milking, as this reduces potential pasture consumption and cow lying time. The robotics system must also work with long walking distances for cows, and the impact of topography and variable weather on cow movement to the parlor. Many NZ farms run crossbred herds resulting in and range of animal sizes, and robotics would need to work in this context. Additionally, there may be a need to manage cows that do not fit the system (Rijswijk et al., 2021).

#### 3.2.2 Workplace design and people

The cost of labor, and ability to source people, will be a continual feature of the agricultural labor market in countries like NZ. There is high turnover among employees, therefore robotics technology must be easy to learn for new staff. In general NZ farms are focused on low farm working costs, and this influences technology investment decisions. A major consideration relates to whether the robotics would be fully autonomous (e.g., like the current AMS) or just focus on cupping cows, therefore requiring one person at the parlor to complete other tasks. This will impact the technology investment required (i.e., the latter option would need to be lower cost), but also will limit farmer’s ability to save on labor to offset investment costs. Having a robot that just attaches cups to cows would still have benefits such as: reduced injuries and physical demands on milking staff, increased job flexibility, ability for a wider range of people (e.g., age, height, strength) to be involved in milking, and creating an innovative image of dairy farming. There may be negative workplace impacts, such as changes to the self-identity of farmers if robotics replaces the role of milking completely.

#### 3.2.3 Farm business structure

Due to investment costs, robotics may impact large and small farms differently in relation to access to capital and technology investment. Additionally, adoption of smart technologies such as high throughput milking robotics may increase farm intensification and expansion (Klerkx et al., 2019). In the NZ system, we identify considerations related to determining who pays for service, support and maintenance as the NZ sector involves both farm owners and sharefarmers with different ability to access finance. Milking technology investments pose challenges for sharefarmers who don’t make fixed capital investments and move farms every 2,3 years on average therefore need highly portable assets.

Robotics will need to be applicable to both early career and late-career farmers with different ICT skill levels and motivations (Christiaensen et al., 2021). For example, early career farmers may have a high interest in technology but limited access to finance, whereas late-career farmers may have access to finance but less motivation to take on long-term investments. Multi-farm ownership structures could impact the level of adoption across the dairy sector. An additional consideration is an assessment of implications of concentration in power with technology providers–e.g., “right to repair” contracts, accessible data, and interoperable systems (Carolan, 2020).

#### 3.2.4 Financial

Financial aspects have been a major limitation on adoption of existing AMS technology in NZ. For novel robotics, a significant barrier will be the uncertainty that banks have related to providing finance. This will relate to not just overall investment costs, and return on investment, but also assessing depreciation and resale values. Farmers and banks look for short (2–4 years) return on investment, particularly where technology uncertainty is high (Rutten et al., 2018), and such timeframes seem unachievable with the current initial high cost of robotic investments. Some technology providers offer subscription models, reducing the entry cost, and sunk costs, for farmers. Return on investment will also relate to the type (cost) of labor the technology replaces. High throughput robotics may be using high cost technology to do low cost jobs, the benefit:cost implications of this need to be considered. Unintended financial consequences also must be considered, such as reduced farm business flexibility where the need to leverage the robotic investment locks farms in to continuing with their current enterprise mix.

#### 3.2.5 Sustainability

To ensure broad sustainability outcomes with milking robotics, a key consideration is the implications for animal well-being through use of the robot. This would relate to aspects such as positive or negative cow:robot interaction (Holloway et al., 2014). Use of robotics and associated sensor equipment may enable opportunities to improve detection of animal health issues. For example, most NZ farmers identify mastitis issues initially through bulk tank somatic cell count, followed by teat stripping (Kamphuis et al., 2016). The use of inline mastitis sensors on NZ farms is currently low (Dela Rue et al., 2020) and wider use of robotics-based sensor systems could lower the time and skill level required to identify such diseases.

#### 3.2.6 Market factors

A major market consideration relates to limited market size for milking robotics in pasture-grazed systems. This market size is further diminished because a 20–30 years replacement cycle for milking parlor infrastructure will limit the number of farmers installing new robotic technology. This will have a large impact on cost-benefit for farmers who may only look to robotics when they need to replace aged infrastructure. Additionally, wide-ranging milking robotics patents may prohibit innovation in the milking robotics domain. Current supply chain settings (farms selling whole milk straight to processor) may limit individual farmers creating value add through robotic technology capabilities.

#### 3.2.7 Social well-being

A major aim of robotics is to substitute for labor, therefore milking robotics could have positive and negative impacts on social well-being. Automated milking on large farms may enable farmers to have more time to connect with their community and family and reduce physical workload enabling older farmers to remain connected with milking tasks. Lower physicality and milking skill requirements through robotics may also enable people from the community to work part time on farms. A reduction in labor on farms may lead to greater farmer isolation, and farms may support fewer jobs in the wider community. Conversely, adoption of robotic technologies on farms could lead to new jobs in the local community for high-skilled technology service and support.

#### 3.2.8 Regulation and policy

NZ is a light-touch regulation economy with few subsidies, but adoption of milking robotics may require some form of investment incentives to. Potential regulatory barriers also need to be assessed in robotics development; such as food safety regulations related to milk harvesting and cooling (Eastwood and Renwick, 2020), and implications under animal care regulations (Manning et al., 2021; Tzachor et al., 2022). A wider consideration is the long-term perception of automated farming among consumers and the wider public (Romera et al., 2020; Pfeiffer et al., 2021).

#### 3.2.9 Knowledge base and networks

Support of the robotics is vital. Milking is time critical, thus service and support networks must be able to respond within short timeframe. Breakdowns of milking equipment have large implications for farmers and cows. Initial support should be provided remotely as rural travel times can be large. Local service agents will need to have specialist skills, and spare parts, to maintain robotics and ICT systems (Carolan, 2020). NZ dairy technology support networks would need upskilling in this regard. More generally there will be changes required regarding the skills of farmer advisory networks (e.g., farm system consultants, nutritionists, vets) (Ingram and Maye, 2020). This would involve developing skills in helping farmers interpret, and make decisions from, the data collected by robotics systems (Eastwood et al., 2019a).

#### 3.2.10 Technology and engineering

There are some specific engineering considerations for design of high throughput milking robotics. Milking cup attachment speed is important, as farmers will look to match what they can achieve manually, however this is a tradeoff with technology cost. Modern imaging systems should be used to improve cup attachment. Farmers may accept extended milking times from slower cupping speed on robotic rotary parlors if they can complete other tasks while supervising the milking process. Robotics must be robust to deal with the wet and dirty operating environment. Ongoing maintenance costs can be an issue for robotics, a successful system must minimize these costs. Maintenance cost structures must also be transparent for farmers prior to purchase. Internet connectivity is an issue on many NZ farms, robotics must be able to work within this constraint. Component technology solutions are becoming tiresome for farmers who operate multiple devices, software systems, and apps (e.g., animal and feed management, compliance, and timesheets) without common log-ins or with data duplication. Robotic systems must easily exchange data from other platforms as required, such as herd management systems or animal health sensors.

#### 3.2.11 Summary of design guide considerations

The framework outlined in this paper proposes a range of design considerations required for an innovation to meet the market needs adequately while being cognizant of socio-ethical and financial factors. For example, while productivity enhancing technologies will become increasingly important in agriculture (Christiaensen et al., 2021), taking a systems view can encourage developers, industry organizations and policy makers not to lose sight of potential impacts on worker satisfaction and well-being in the longer term. While the focus of technology development is often on replacing current manual tasks through retrofitting robotics into existing systems (Prause, 2021), taking a broader approach to understanding the pain points may lead us to redesign systems around future robotic opportunities.

Following the design guide factors enabled us to identify specific characteristics to consider for novel milking robotics. Their holistic nature can also be a means to breaking out of conventional thinking around technology. One consideration identified was that in pasture-grazed systems, milking robotics may not completely replace the labor associated with milking, a finding backed up by Prause (2021). For example, the parlor could be overseen by a milking manager, who does not actively attach cups and can undertake other jobs during the milking period, for example data analytics. This changes the dynamics of the return-on-investment calculation for farmers, as less labor would be saved, but also changes the design specifications for the robot itself, as 100% accuracy may not be required due to the presence of the milking manager to attend to unsuccessful robotic cupping attempts or other minor issues. Another outcome from using the design guide related to the potential to reimagine pasture-grazed farm systems by integrating milking robotics in a “mission-led” approach, for example improving workforce productivity by 25%. This might involve not only the use of novel robotics, but also system changes such as once-a-day milking, and virtual fencing, to “stack” different approaches that enable farms to operate with fewer people. The guide then prompts us to ask subsequent questions about the impacts of such a systemic change on other socio-ethical issues such as farmer identity, employee satisfaction, rural community impacts and consumer perceptions of farming (Holloway et al., 2014; Romera et al., 2020; Pfeiffer et al., 2021).

## 4 Conclusion

The case study analysis highlighted potential use of a design guide approach to robotics and automation innovation development. In practice, effective development of holistic design guides must be underpinned by co-design processes that involve the end-users as well as other relevant stakeholders such as advisors and consumers. Work by authors such as Ditzler and Driessen (2022) and Romera et al. (2020) have highlighted such processes in approaching system redesign and technology integration with complex farming systems. The design guide we have proposed could form the basis of discussions with end-users (farmers and employees) and technology designers and manufacturers, with the goal of iterating and custom fitting the design characteristics. Co-design would also have the benefit of initiating conversations focused on identifying both technology and farm systems innovations that could enable successful implementation of robotics on future dairy farms.

Finally, design guides could be used as a tool for enacting the RRI principles in development of agricultural robotics. Our proposed guide was based on a holistic range of factors identified through a range of published studies. This holistic guide aligns with the concept introduced by (Rijswijk et al., 2021) of “socio-cyber-physical systems” where the interactions between social, cyber, and physical components of smart farming can be assessed. This process can therefore enable greater *inclusion* of perspectives of actors and stakeholders related to robotic systems and is a tool for *anticipating* positive and negative implications of robotic development and use on-farm.

## Data Availability

The original contributions presented in the study are included in the article/Supplementary Material, further inquiries can be directed to the corresponding author.
